# Low incidence of SNVs and indels in trio genomes of Cas9-mediated multiplex edited sheep

**DOI:** 10.1186/s12864-018-4712-z

**Published:** 2018-05-25

**Authors:** Xiaolong Wang, Jing Liu, Yiyuan Niu, Yan Li, Shiwei Zhou, Chao Li, Baohua Ma, Qifang Kou, Bjoern Petersen, Tad Sonstegard, Xingxu Huang, Yu Jiang, Yulin Chen

**Affiliations:** 1 College of Animal Science and Technology, Yangling, 712100 China; 2 College of Veterinary Medicine, Northwest A&F University, Yangling, 712100 China; 3 Ningxia Tianyuan Tan Sheep Farm, Hongsibu, 751999 China; 4 Institute of Farm Animal Genetics, Friedrich-Loeffler-Institut, 31535 Neustadt, Germany; 5 Recombinetics, St. Paul, Minneapolis, MN 55104 USA; 6 School of Life Science and Technology, ShanghaiTech University, Shanghai, 201210 China

**Keywords:** Genome editing, CRISPR/Cas9, Whole genome sequencing, Off-target; mutation rate, Sheep

## Abstract

**Background:**

The simplicity of the CRISPR/Cas9 system has enabled its widespread applications in generating animal models, functional genomic screening and in treating genetic and infectious diseases. However, unintended mutations produced by off-target CRISPR/Cas9 nuclease activity may lead to negative consequences. Especially, a very recent study found that gene editing can introduce hundreds of unintended mutations into the genome, and have attracted wide attention.

**Results:**

To address the off-target concerns, urgent characterization of the CRISPR/Cas9-mediated off-target mutagenesis is highly anticipated. Here we took advantage of our previously generated gene-edited sheep and performed family trio-based whole genome sequencing which is capable of discriminating variants in the edited progenies that are inherited, naturally generated, or induced by genetic modification. Three family trios were re-sequenced at a high average depth of genomic coverage (~ 25.8×). After developing a pipeline to comprehensively analyze the sequence data for de novo single nucleotide variants, indels and structural variations from the genome; we only found a single unintended event in the form of a 2.4 kb inversion induced by site-specific double-strand breaks between two sgRNA targeting sites at the *MSTN* locus with a low incidence.

**Conclusions:**

We provide the first report on the fidelity of CRISPR-based modification for sheep genomes targeted simultaneously for gene breaks at three coding sequence locations. The trio-based sequencing approach revealed almost negligible off-target modifications, providing timely evidences of the safe application of genome editing in vivo with CRISPR/Cas9.

**Electronic supplementary material:**

The online version of this article (10.1186/s12864-018-4712-z) contains supplementary material, which is available to authorized users.

## Background

The type II bacterial clustered, regularly interspaced, palindromic repeats (CRISPR)-associated (Cas) system is a powerful and versatile genome editing tool, which has already been tested in the genomes of large animal models [1–4]. This system consists of a single polypeptide endonuclease Cas9 complexed with a single guide RNA (sgRNA) that allows complementation for up to 20 nucleotides of target DNA sequence. It is not uncommon for the relatively short target recognition requirement to allow unwanted off-target mutations, because of potential for non-unique matching and allowance of sequence mismatches distal from the PAM sequences at the 5′ end of sgRNAs.

Off-target effects of the CRISPR/Cas9 system have raised serious concerns about its ultimate applicability. Schaefer et al. previously reported that gene editing can introduce hundreds of unintended mutations into the genome, and have attracted wide attention. This conclusion resulted from sequencing two entire genomes of mice that had undergone CRISPR-mediated gene editing and an untreated control mouse genome [5]. However, since only one control animal, one guide RNA and a single CRISPR-vector for DNA injection were used [5, 6], the experiment design has very limited power to show that the magnitude of off-target effects is unusually high. However, this conclusion has been recently corrected by themselves when using additional mouse lines for further whole genome sequencing (WGS) analyses [7].

WGS provides a better dataset to fully characterize the mutation profiles at genome-wide scale [8, 9], compared with Sanger or short read deep sequencing of target and predicted off-target PCR amplicons. Furthermore, WGS data can be used to comprehensively look at sequence nucleotide variants (SNV), smaller (< 100 bp) insertion/deletions (indels), and structural variants (SVs) (e.g. inversions and translocations) [10]). In the present study, we used the genetically modified sheep generated through multiplex injection of six sgRNAs targeting three genes (*MSTN*, *ASIP*, and *BCO2*) and Cas9 mRNA in one-cell-stage embryos by previous studies [11, 12].

Take advantage of family trio-based WGS which is capable of discriminating variants in the edited progenies that are inherited, naturally generated, or induced by genetic modification, we went back and completed comparative mutational analyses of WGS from three trios (founder animals with edits and their parents). We found that the rate of de novo mutations in the edited animals was equivalent to the mutation rate in human studies, suggesting the de novo SNVs were rather resulted from normal spontaneous mutagenesis, and not induced by genetic modification.

We herein address the concerns about the unintended off-target mutations in CRISPR/Cas9-mediated edited animals, and provide evidence to show that the incidence of off-target mutations in large animals generated by CRISPR/Cas9 system may be sufficiently low enough to apply this technology for generating viable animals.

## Results

### Analyses of WGS family data

**Fig. 1:**
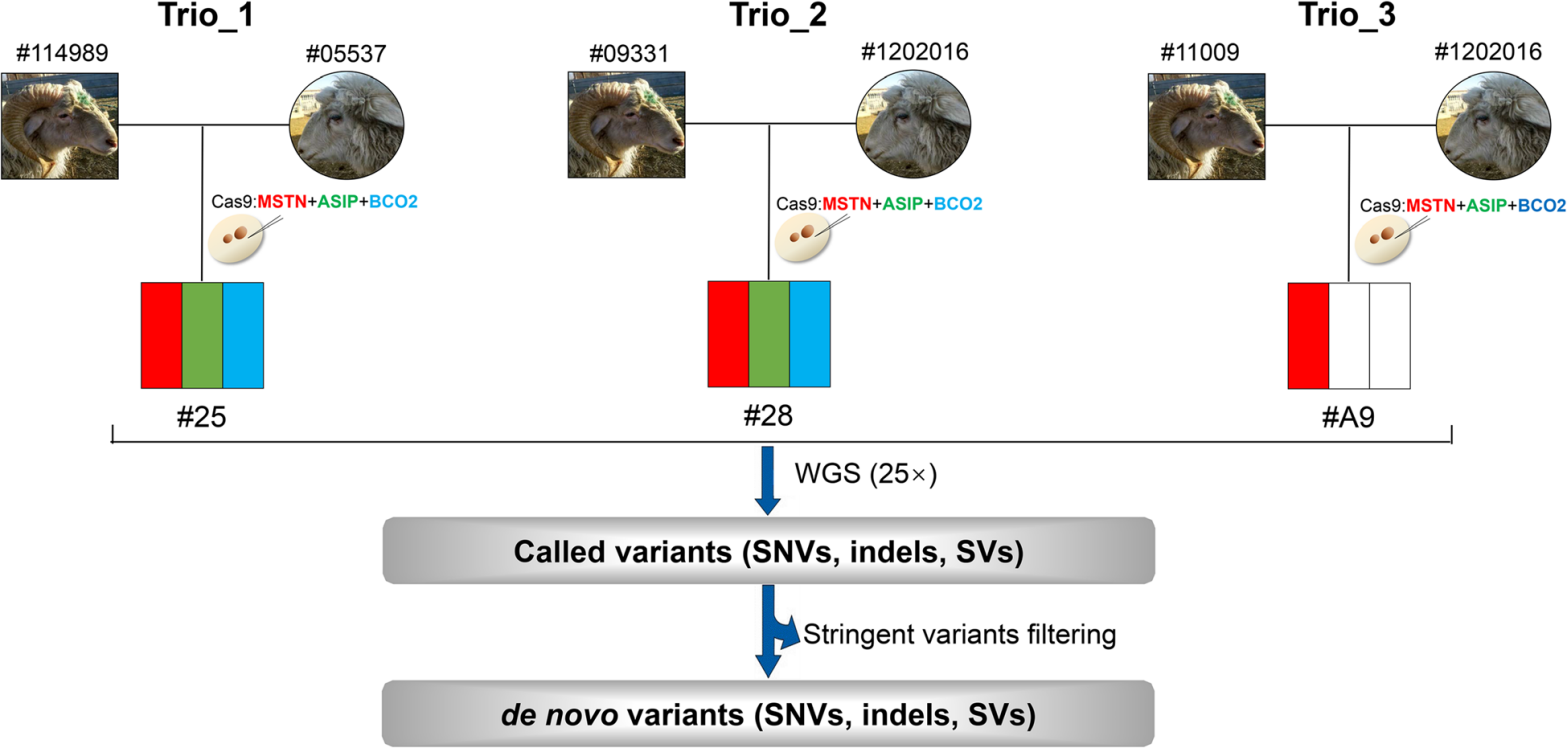
Outline of identifying de novo variants that may be induced by genetic modification through WGS of three family trios in sheep. #25, # 28, and #A9 are gene-edited animals that generated by CRISPR/Cas9, colors in the founder animals indicated the information of targeted genes

Cas9 mRNA and gRNAs for three functional gene targets (*MSTN*, *ASIP*, and *BCO2*) were multiplex-injected into one-cell-stage sheep embryos, to produce animals with gene disruptions affecting observable phenotypes for muscling, coat color and type [11]. We selected three family trios for founder animals #25, #28, and #A9 for WGS variant analyses. The edited genotypes of these founders are summarized in Fig. 1 as not all founders were successfully edited at all three target sites. Specifically, genetic modification occurred in all the three genes for #28, at *ASIP* and *BCO2* loci for #25, and only at *MSTN* for #A9.

WGS of three trios animal genomic DNAs yielded a total of 550 Gb of raw data, and produced between 460 and 566 million sequence reads per animal (Additional file 1: Table S1). Over 99.34% of the generated sequence reads were mapped, indicating that high quality sequences were obtained. Sequencing depth per animal ranged from 23.08–28.87× genome coverage after alignment to the sheep reference genome (Oar v3.1) [13], which resulted in identification of 21,086,636 SNVs.

### Identification and validation of de novo SNVs

**Fig. 2:**
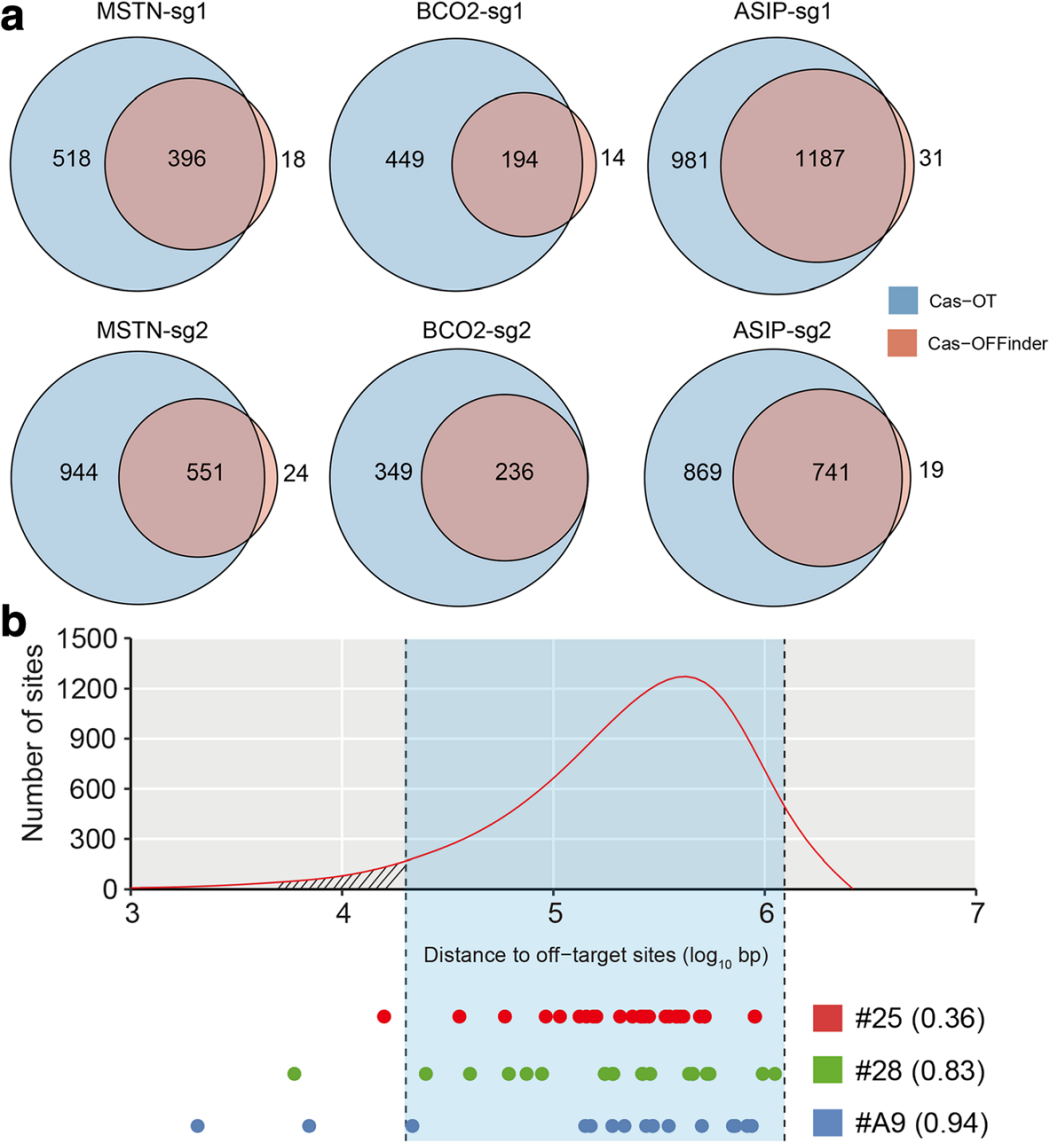
Identification of de novo SNVs through WGS of three family trios that include edited sheep. (**a**) The predicted off-target sites by CasOT and Cas-OFFinder at each target site. (**b**) The distances between 100,000 randomly selected sites (upper), and de novo SNVs (below) to predicted off-target sites. The off-target sites were defined as 1 mismatch at seed region, and up to 4 mismatch at non-seed region. The least distance to predicted off-target sites was chosen. The area between two dashlines represents the 95% confidence interval

To detect mutations that may be derived from Cas9-mediated genetic modification, a series of stringent variant filtering procedures were completed (Fig. 2a). The completion of a series of stringent variant filtering procedures, combined with validation with Sanger sequencing using all trio members and the offspring of #28, we show that only ~ 20 de novo SNVs remained in each edited animal (Table 1). Specifically, 710, 1561, and 632 SNVs were identified after SNV calling, quality check, and filtering SNVs that existed in our sheep SNP database (294 genomes from 74 breeds, > 79 million SNPs, unpublished data). To verify the existence of these SNVs, we randomly selected 20 SNVs for each founder animal, and sequenced all trio members by Sanger sequencing. We could only validate 9–30% of the SNVs (4/13, 1/11, 2/12) in each founder (Table 1). We then conducted stringent filtering procedures including read depth, phred-scaled likelihood (PL) scores [14], and manual examination to show only 26, 20, and 16 SNVs remained as unique in each founder. Sanger sequencing confirmed that most of these SNVs existed and were not inherited from their parents (Additional file 1: Figure S1), suggesting the pipeline was robust for identification of de novo SNVs. In addition, we sequenced the edited sites and 6 randomly selected SNVs in the offspring (*n* = 3) of founder animal #28 (Additional file 1: Figure S2 and S3), to confirm that Cas9-mediated mutations and nature de novo mutations were both transmittable at the germline level [15].

**Table 1:** Summary of SNVs called in the founder animals of three trios by WGS

SNVs	#25	#28	#A9
Called by GATK	21,086,636		
Founder specific SNV which were also identified by Samtools	3430	5294	3288
Filtering SNVs existed in the sheep SNP databases (*n* = 294, > 79 million SNPs)	3383	5252	3252
Filtering SNVs have not existed in the sheep SNP databases	2227	2696	2355
Filtering SNVs existed in the reads with alternative alleles (< 25%), and the read depth in founders < 3 within a trio	710	1561	632
Confirmed SNVs by Sanger sequencing	(4/13)	(1/11)	(2/12)
The PL scores for each genotype > 20, 0 and > 0, the PL scores for each genotype in both parents should be 0, > 20 and > 20	130	642	80
Filtering SNVs uncovered by both forward and reverse reads	82	313	52
Manual examination	26	20	16
Confirmed SNVs by Sanger sequencing	(20/22)	(8/9)	(10/11)

Tsai reported that less overlapped off-targets were obtained when using different computational tools [16]. We then predicted the potential off-target sites with two computational programs, CasOT [17] and Cas-OFFinder [18]. We found that, under the same filtering parameters (up to 5 mis-matches [19]), the Cas-OFFinder is able to identify much more off-targets than the CasOT program (Fig. 2b). We also found over half of the off-targets (53.6%) identified by CasOT can be found in the off-target sites predicted by Cas-OFFinder (Fig. 2a). More importantly, we simulated the distance between 100,000 randomly selected sites, and de novo SNVs to the potential off-target sites that predicted both with CasOT [17] and Cas-OFFinder [18] (Additional file 2: Table S2), no significant effects were observed between the random selected sites and de novo SNVs (Fig. 2b). These results highlighted that de novo SNVs were rather the result of normal spontaneous mutagenesis, and not induced by genetic modification.

### Identification and validation of de novo indels and SVs

Next, we did a comparative analysis within each trio to search for Cas9-induced small indels and SVs, given the possible likely outcomes of Cas9 induced double-strand breaks (DSB) repaired via non-homologous end-joining (NHEJ). After stringent indel filtering procedures including read depth, PL value and manual examination, 25, 26, and 17 indels were determined as candidate de novo indels in each founder animal (Table 2). Besides the five edited indel sites confirmed by sequencing (Additional file 1: Figure S4), we also validated several indels for each trio (Additional file 2: Table S2).

**Table 2:** Summary of indels and SVs called in the founder animals of three trios by WGS

Variants	#25	#28	#A9
*Indels*			
Called by GATK+Samtools	2,225,916		
SNVs that specific for each founder and filtering SNVs existed in the reads with alternative alleles (< 25%), and the read depth in founders < 3 within a trio	57	64	40
The PL scores for each genotype in founders should be > 20, 0 and > 0, the PL scores for each genotype in both parents should be 0, > 20 and > 20	56	58	39
Manual examination to remove mis-alignment or miscall INDELs	25	26	17
*SVs*			
(1) Called by BreakDancer	2491	3198	3348
After removal of common SVs in every two founders, and the read depth < 50%	8	3	15
(2) Called by Lumpy	109	94	132
After removal of scaffold SVs	3	4	1
(3) Called by CNVcaller	1547	1124	1144
Candidate de novo CNVs	6	3	4

**Fig. 3:**
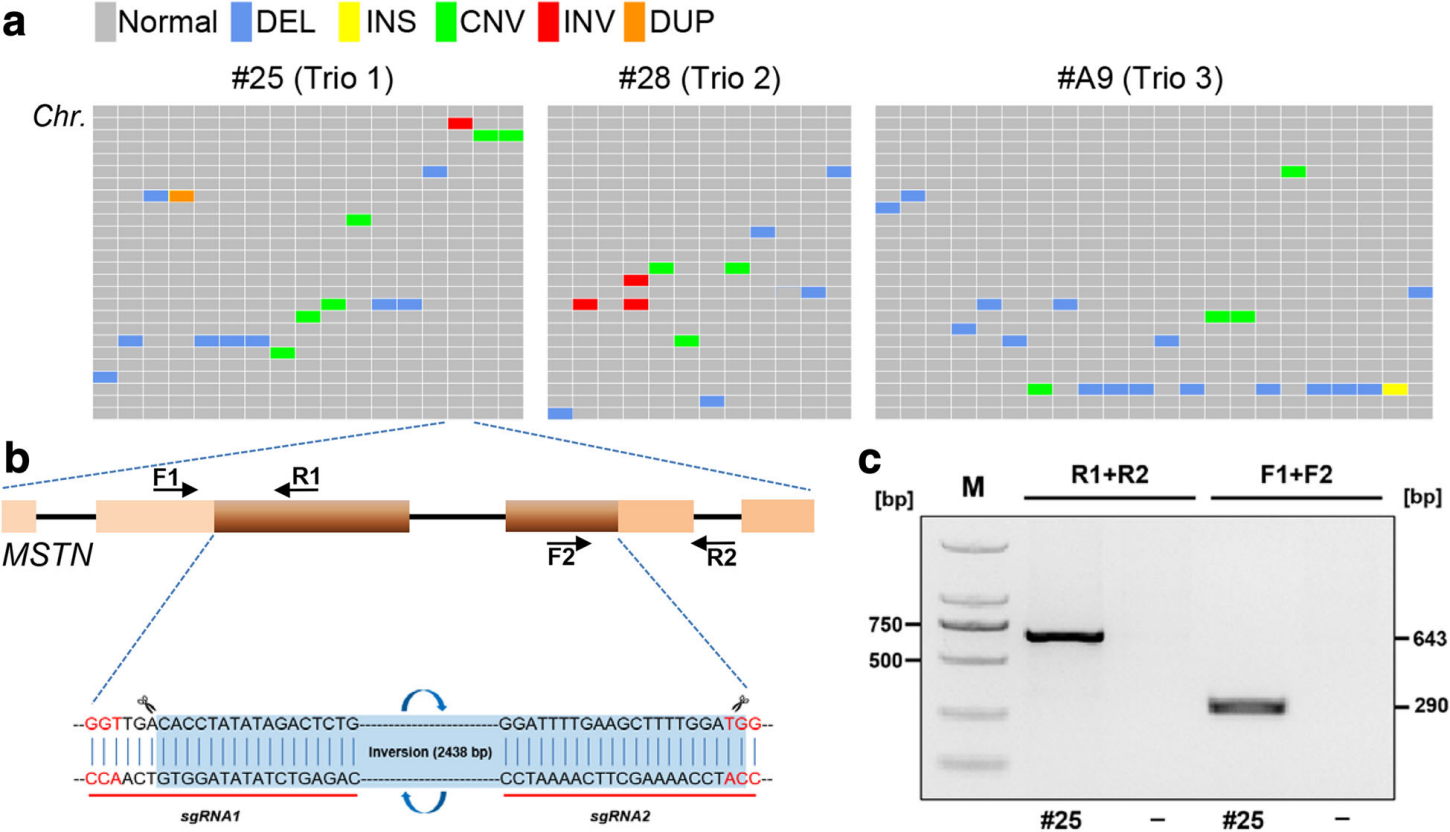
Validation of the 2.4 kb inversion at *MSTN* locus in founder #25. (**a**) Schematic distribution of SVs in the edited animals. (**b**) Diagram of the *MSTN* gene targeted by CRISPR/Cas9 with two sgRNAs. The sgRNA-targeting sequences are highlighted. The inversion region is marked with light blue background. The protospacer adjacent motif (PAM) sequences of NGG are highlighted in red. The positions of the forward and reverse PCR primers used for PCR amplification are indicated by arrows. (**c**) PCR results confirmed predicted inversion of targeted region at *MSTN* locus in founder #25

We then searched for large-scale genomic alterations induced by Cas9. We generated a short list of candidate SVs in each trio (Fig. 3a, Table 2, Additional file 2: Table S2), none of them were close or within the edited sites, except for a single inversion found at *MSTN* in edited animal #25. This 2.4 kb inversion resides between the two DSBs induced by Cas9 among 8 bp downstream and 2 bp upstream of the PAM sites, and perfectly fit with the gap between sg1 and sg2 sites (2438 bp in length) (Fig. 3b). We further verified the existence of this inversion in #25 by amplification of the upstream and downstream junction sequences (Fig. 3c), we assume that this inversion was induced by simultaneous site-specific DSB at the sg1 and sg2 sites. However, there was no visual or clinical evidence that genetic modification caused any abnormality for founder #25 (both *ASIP* and *BCO2* disrupted) so far (2 years old), it seems that the inversion did not influence animal’s health. Nonetheless, we checked for this inversion occurred in other founders obtained through genome editing (*n* = 54) and found no other evidence of this event (Additional file 1: Figure S6).

Taken together, our WGS analyses findings for de novo variants indicates that Cas9-induced genetic modifications did not induce significant off-target alterations at the whole-genome level for 3 of our sampled 54 founder animals. Furthermore, our results suggested that across sequence variant type all the de novo variants except one were randomly accumulated during parental gamete or embryonic development and were not the consequences of off-target activities that were induced by Cas9 modification.

## Discussion

Recent studies have demonstrated that the type II CRISPR-Cas9 system of *Streptococcus pyogenes* holds great promise to modify gene variants that express valuable traits in a wide variety of crops and animals [20, 21]. We previously show that site-specific gene modification can be efficiently achieved by co-injection of Cas9 mRNA and sgRNAs that target multiple genes in sheep [11]. The integrity of the resultant modified genomes may have some heightened biosafety concerns from regulators and producers; however, our data unequivocally shows what would be predicted. The rate of natural de novo mutations for sequence variants is more than an order of magnitude higher than unintended off-target mutations.

This study represents the first comprehensive trio-based WGS effort to identify genome-wide de novo variants through a filtering pipeline which includes using the largest sheep sequence data so far. We show the power of WGS in the identification of structural genomic changes which cannot be recognized by PCR-base method (e.g. PCR-RFLP or Sanger sequencing). We were able to identify 26, 20, and 16 germline de novo SNVs in these three founder animals from each trio respectively, the estimated germline de novo SNV rate (1.36 × 10^− 8^) in the present study, which was equivalent to the germline de novo mutation rate in parent-offspring trios in humans (1.17 × 10^− 8^ in CEU; 0.97 × 10^− 8^ in YRI) [22–24] and a large cattle population (1.20 × 10^− 8^) [25]. These results indicated that the de novo SNVs in the founder animals are generated naturally rather than induced by genetic modification. Our study adds important new perspectives to the recently reported results by Schaefer et al. [5], in which the unintended mutations in two mutant animals are likely to have preexisted prior to injection or were caused by residual, overexpression of Cas9 from the injected vector.

Through deep sequencing which is able to fully characterize the mutational profiles [8, 9], combined with Sanger sequencing validation using all trio members and the offspring of the founder animal #28, we found that off-target mutations that could be attributable to the nucleases in the founders within these three trios are rare. We therefore provide primary support that Cas9-mediated gene modification induced little or no off-target mutations in the present study, consistent with the recent reports of low incidence of CRISPR/Cas9-mediated off-target mutations in the human cells [26, 27] or mice [28, 29]. Although a 2.4 kb inversion was identified in the founder animal #25, which presumably due to DB-induced cleavage at two sgRNA target sites, the proportion of this inversion is rare when performing multiplex genetic manipulation in sheep genome (1.85%, 1 out of 54 individuals).

On the other hand, Cas9-induced DSB provide an alternative approach to achieve precise inversions or duplications of DNA fragments using two or more sgRNAs in manipulating mammalian genomes [30, 31]. Because the occurrence of off-target effects is one of the crucial issues in the use of genome editing in agriculture [32], our results indicated we could generate animals with no detectable off-target mutations, and further confirmed the reliability of a multiplex-based CRISPR/Cas9 approach for the production of gene-edited animals.

## Conclusions

In summary, we present here the first report of characterizing candidate de novo mutations through trio-based re-sequencing in sheep. Those identified de novo mutations are highly important to advance the understanding off-target modifications in genetic modified animals produced by zygote injection, and thereby providing primary results to evaluate the bio-safety of gene-edited animals.

## Methods

### Animals and pedigree information

The sheep used in the present study were maintained at the Ningxia Tianyuan Sheep Farm, Hongsibu, Ningxia Autonomous Region, China. Since we transferred pooled embryos which were derived from multiple ewes into surrogates previously [11], we did paternity test to identify family members for the selected three trios. The pedigree information of these three family trios was determined using nine microsatellite markers (BM4621, BM143, OarHH55, OARJMP8, BM6438, BM6506, BM1824, OarDB6, and ILSTS004) reported previously [33]. All protocols involving the use of animals were approved by the College of Animal Science and Technology, Northwest A&F University (Approval ID: 2014ZX08008002).

### Whole-genome sequencing

Individuals from three family trios comprising three founders (#25, #28, #A9) were selected for WGS (Additional file 1: Table S1). Genomic DNA was extracted from blood samples (live animals) or ear tissues (stillbirth) using a Qiagen DNeasy Blood and Tissue Kit (Qiagen). For construction of the WGS library, 1 μg of gDNA was fragmented to around 300 bp by ultrasonication using a Covaris S2 system. Then, the sheared DNA fragments were used for library construction using an Illumina TrueSeq DNA library preparation kit. The final quality-ensured libraries were sequenced on an Illumina Hiseq2500 for 125-bp paired-end reads. All the reads that passed the quality control were converted into fastq files.

### Identification of de novo variants

All of the reads for each sample were mapped to the sheep reference sequence assembly v3.1 [13] using BWA tools [34]. Local realignment and base quality re-calibration were performed using GATK (v.2.7–2) [35]. The raw sequencing reads were first filtered to remove low quality paired reads with the following criteria: (1) reads with > 10% N bases; (2) reads with > 50% bases with a sequencing quality of < 5; (3) reads with residual length of < 60 bases after the adaptor sequences were trimmed. Both SNVs and indels (2–50 bp) were called using GATK ‘IndelRealigner’ [35] and Samtools [36]. Additionally, SNVs and indels were separately re-calibrated as described in GATK Best Practices, and quality filters (quality score < 50.0, reads coverage> 300) were applied.

Putative de novo SNVs for each founder were extracted according to the following criteria: (1) SNVs that specific for each founder and were also identified by the samtools; (2) filtering SNVs that are existed in the sheep SNP databases (*n* = 294, > 79 million SNPs, unpublished data) only with alternative alleles; and filtering SNVs that did not exist in the ovine SNP databases; (3) filtering SNVs that were existed in the reads with alternative alleles (< 25%), and the read depth in founders < 3 within a trio [14]; (4) the normalized, PL scores for the genotypes (AA, AB and BB) of founders (A, reference allele; B, alternate allele), the PL scores for each genotype in founders should be > 20, 0 and > 0, the PL scores for each genotype in both parents should be 0, > 20 and > 20 [14]; (5) the candidate SNVs should be covered with both forward and reverse reads; (6) manual examination was performed to remove mis-aligned or miscalled SNVs. The filtering procedure of indels was same as the extracting of de novo SNVs. SVs were independently detected using BreakDancer [37], LUMPY [38], and CNVcaller [39]. The detailed filtering pipelines for de novo variants are summarized in Table 1.

We searched for putative off-target sites in the sheep genome that might be recognized these six sgRNAs for the *MSTN*, *ASIP*, and *BCO2* genes using CasOT [17] and Cas-OFFinder [18]. The number of predicted off-target sites were summaries in Additional file 1: Table S2, and we defined the potential off-target sites as 1 mis-match at seed regions, and 4 mis-matches at non-seed regions, according to Boyle et al. [19].

### Validation of indels and SNVs in edited animals

The offspring (#171001, #171004, and #171018) of edited animal #28 were generated by natural mating #28 with three wild-type ewes (#1500442, #1500444, #1500510). Sanger sequencing was conducted to verify the regions with genetic modifications, which were identified by WGS. Purification of PCR products, T7E1 cleavage assay, and Sanger sequencing were performed according to Wang et al. [11]. De novo SNVs were selected for validation by Sanger sequencing. Primers that encompassing these SNVs were designed (Additional file 2: Table S2), and were used for PCR amplification. The Integrative Genomics Viewer (IGV) browser was used to visualize the sequence reads and indels on target sites [40].

## Additional files

Additional file 1: Supporting figures and tables. **Figure S1.** Sanger sequencing confirms the SNVs identified by WGS in three trios. Example of SNVs sequenced in trio members. **Figure S2.** Sanger sequencing validation of the genetic modification SNVs in the offspring of #28 (#171001, #171004, and #171018). Target sequences complementary to sgRNAs of targeted genes are in red text, while the PAM sequences are marked in green. The mutations are marked in blue, dashlines indicate deletions, and lowercases indicate insertions or replacements. Deletions (−) and mutations (m) are shown to the right of each allele. The genotypes are shown to the right with the rates of total clones for TA-sequencing. **Figure S3**. Sanger sequencing validation of the genotypes of six SNVs in the offspring of #28 (#171001, #171004, and #171018). **Figure S4**. Sanger sequencing confirms the edited sites (indels) in each gene identified by WGS in three trios. The upper windows were generated by using Integrative Genomics Viewer (IGV) browser (http://software.broadinstitute.org/software/igv/). Target sequences complementary to sgRNAs of targeted genes are in red text, while the PAM sequences are marked in green. The mutations are marked in blue, dashlines indicate deletions, and lowercases indicate insertions or replacements. **Figure S5**. Genome-wide distribution of putative off-target sites in the three trios used for WGS. Putative off-target sites were identified by aligning the sgRNA sequences to the sheep reference genome (Oar v3.1) allowing for a maximum of five mismatches. Potential off-target sites predicted by both Cas-OT and Cas-OFFinder are displayed using the OmiCircos tool (http://bioconductor.org/packages/release/bioc/html/OmicCircos.html). De novo indels in the founder animals #25 (A), #28 (B), and #A9 (C). Putative off-target sites in ASIP (a), BCO2 (b), and MSTN (c). The position of three targeted genes was highlighted with black dots. **Figure S6**. Validation of the 2.4 kb inversion in 54 animals. The founder animal #25 was marked with red color. **Table S1** Detailed information of the three trios for whole-genome sequencing. (DOCX 1633 kb)

Additional file 2: **Table S2.** Summary of predicted of off-targets and de novo variants. (XLSX 587 kb)

## Abbreviations

ASIP: Agouti signaling protein
BCO2: beta-carotene oxygenase 2
BW: body weight
CNV: copy number variations
CRISPR: clustered regulatory interspaced short palindromic repeats
DSB: DNA double-strand breaks
GAPDH: Glycerol phosphate dehydrogenase gene
H&E: hematoxylin-eosin
indels: insertions or deletions
MSTN: myostatin
MUT: mutation
NHEJ: non-homologous end-joining
PAM: Protospacer adjacent motif
PL: phred-scaled likelihood
sgRNA: single guide RNA
SNV: single-nucleotide variants
SV: structure variations
T7E1: T7 endonuclease 1
TALENs: transcription activator-like effector nucleases
TEM: Transmission electron microscopy
WGS: Whole-genome sequencing
WT: wild type
ZFNs: zinc finger nucleases
