# Supplementary material for: Low incidence of SNVs and indels in trio genomes of Cas9-mediated multiplex edited sheep
Source: BMC Genomics. 2018 May 25;19:397. doi: 10.1186/s12864-018-4712-z (PMC5970491; doi:10.1186/s12864-018-4712-z)
Supplement: Supplementary file 1 — Supporting figures and tables. Figure S1. Sanger sequencing confirms the SNVs identified by WGS in three trios. Example of SNVs sequenced in trio members. Figure S2. Sanger sequencing validation of the genetic modification SNVs in the offspring of #28 (#171001, #171004, and #171018). Target sequences complementary to sgRNAs of targeted genes are in red text, while the PAM sequences are marked in green. The mutations are marked in blue, dashlines indicate deletions, and lowercases indicate insertions or replacements. Deletions (−) and mutations (m) are shown to the right of each allele. The genotypes are shown to the right with the rates of total clones for TA-sequencing. Figure S3. Sanger sequencing validation of the genotypes of six SNVs in the offspring of #28 (#171001, #171004, and #171018). Figure S4. Sanger sequencing confirms the edited sites (indels) in each gene identified by WGS in three trios. The upper windows were generated by using Integrative Genomics Viewer (IGV) browser (http://software.broadinstitute.org/software/igv/). Target sequences complementary to sgRNAs of targeted genes are in red text, while the PAM sequences are marked in green. The mutations are marked in blue, dashlines indicate deletions, and lowercases indicate insertions or replacements. Figure S5. Genome-wide distribution of putative off-target sites in the three trios used for WGS. Putative off-target sites were identified by aligning the sgRNA sequences to the sheep reference genome (Oar v3.1) allowing for a maximum of five mismatches. Potential off-target sites predicted by both Cas-OT and Cas-OFFinder are displayed using the OmiCircos tool (http://bioconductor.org/packages/release/bioc/html/OmicCircos.html). De novo indels in the founder animals #25 (A), #28 (B), and #A9 (C). Putative off-target sites in ASIP (a), BCO2 (b), and MSTN (c). The position of three targeted genes was highlighted with black dots. Figure S6. Validation of the 2.4 kb inversion in 54 animals. The founder a [file 12864_2018_4712_MOESM1_ESM.docx]

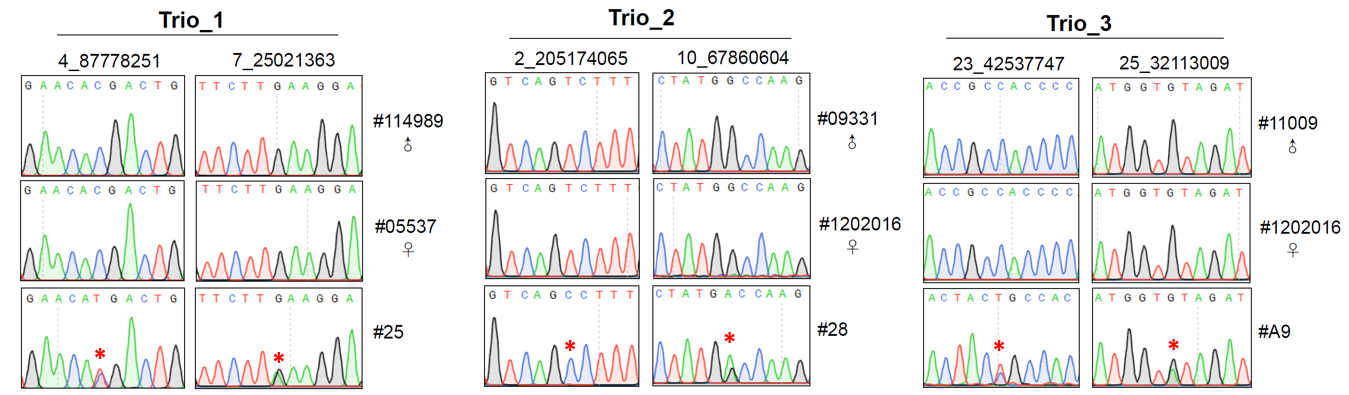
**Figure S1.** Sanger sequencing confirms the SNVs identified by WGS in three trios. Example of SNVs sequenced in trio members.


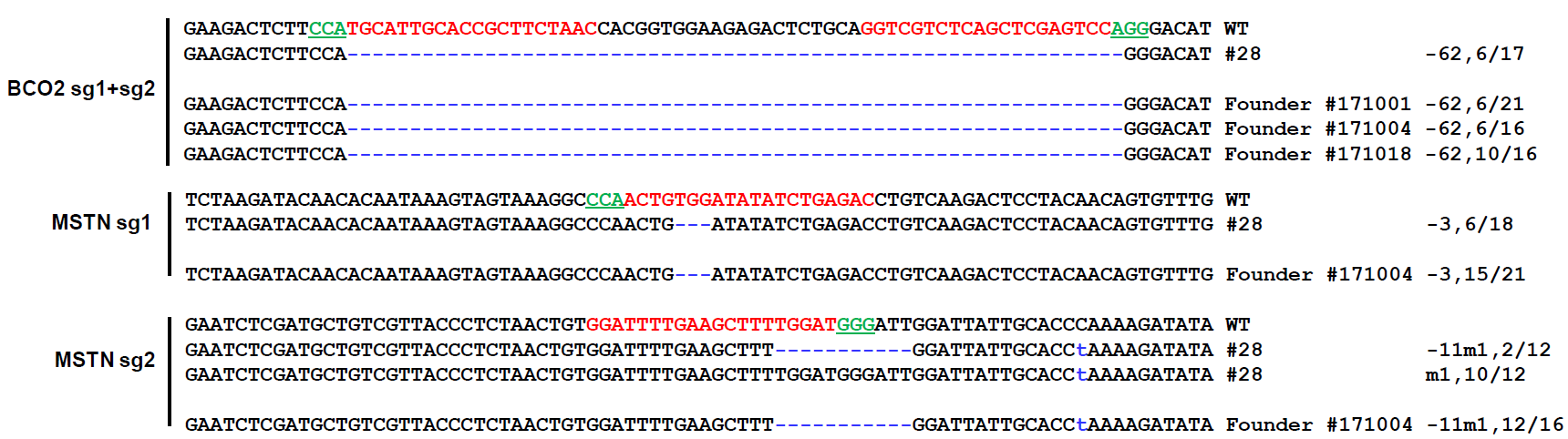
**Figure S2.** Sanger sequencing validation of the genetic modification SNVs in the offspring of #28 (#171001, #171004, and #171018). Target sequences complementary to sgRNAs of targeted genes are in red text, while the PAM sequences are marked in green. The mutations are marked in blue, dashlines indicate deletions, and lowercases indicate insertions or replacements. Deletions (−) and mutations (m) are shown to the right of each allele. The genotypes are shown to the right with the rates of total clones for TA-sequencing.


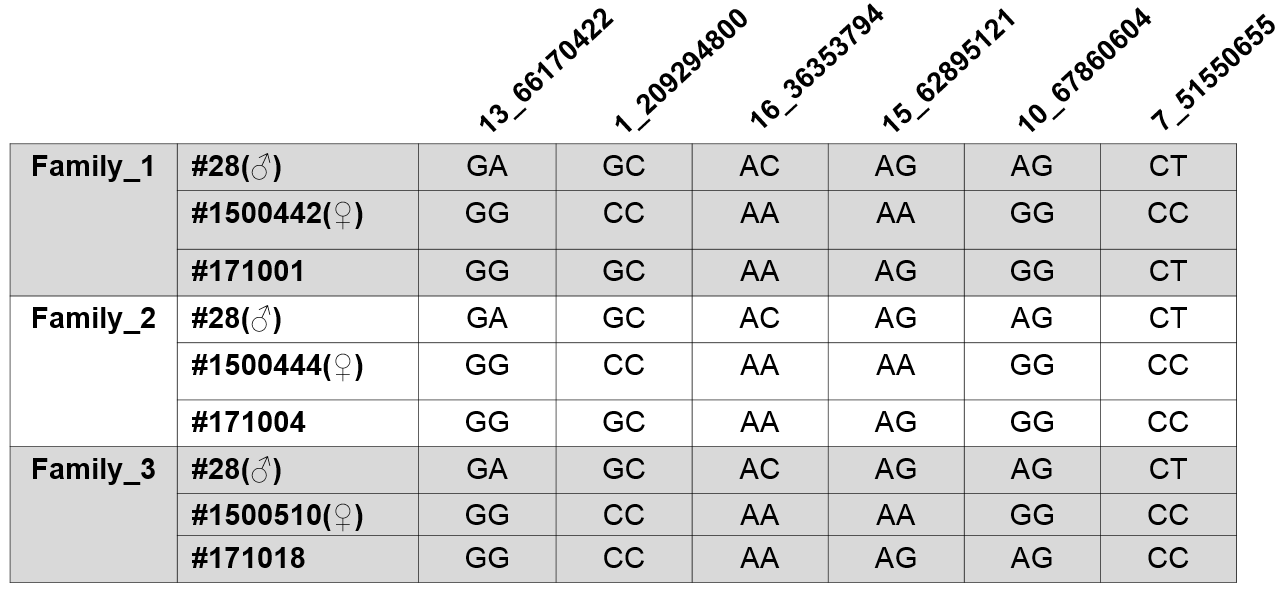
**Figure S3.** Sanger sequencing validation of the genotypes of six SNVs in the offspring of #28 (#171001, #171004, and #171018).


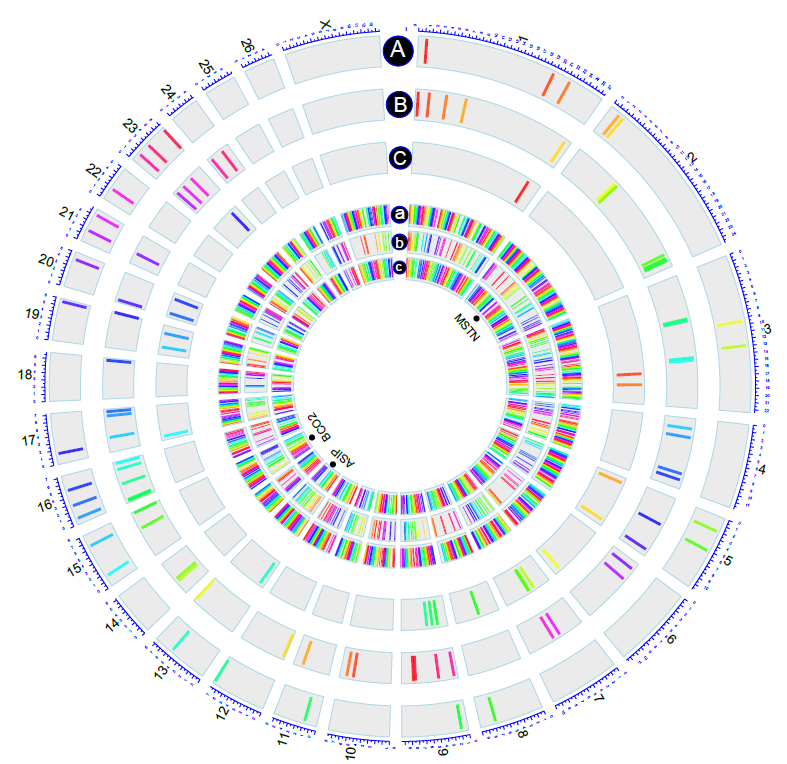


**Figure S4.** Genome-wide distribution of putative off-target sites in the three trios used for WGS. Putative off-target sites were identified by aligning the sgRNA sequences to the sheep reference genome (Oar v3.1) allowing for a maximum of five mismatches. Potential off-target sites predicted by both Cas-OT and Cas-OFFinder are displayed using the OmiCircos tool (http://bioconductor.org/packages/release/bioc/html/OmicCircos.html). *De novo* indels in the founder animals #25 (A), #28 (B), and #A9 (C). Putative off-target sites in *ASIP* (a), *BCO2* (b), and *MSTN* (c). The position of three targeted genes was highlighted with black dots.

**
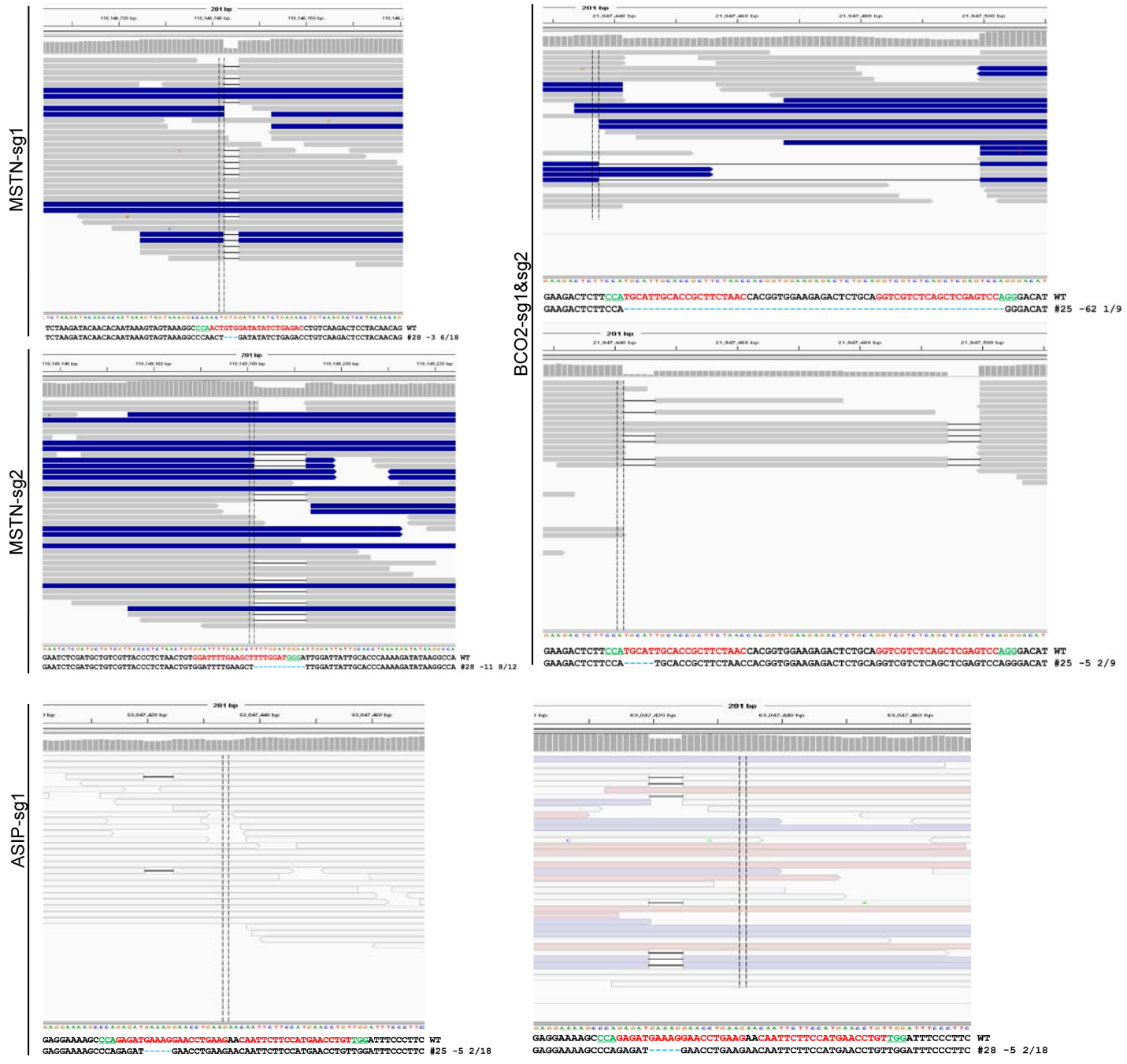
**

**Figure S5.** Sanger sequencing confirms the edited sites (indels) in each gene identified by WGS in three trios. The upper windows were generated by using Integrative Genomics Viewer (IGV) browser (http://software.broadinstitute.org/software/igv/). Target sequences complementary to sgRNAs of targeted genes are in red text, while the PAM sequences are marked in green. The mutations are marked in blue, dashlines indicate deletions, and lowercases indicate insertions or replacements.


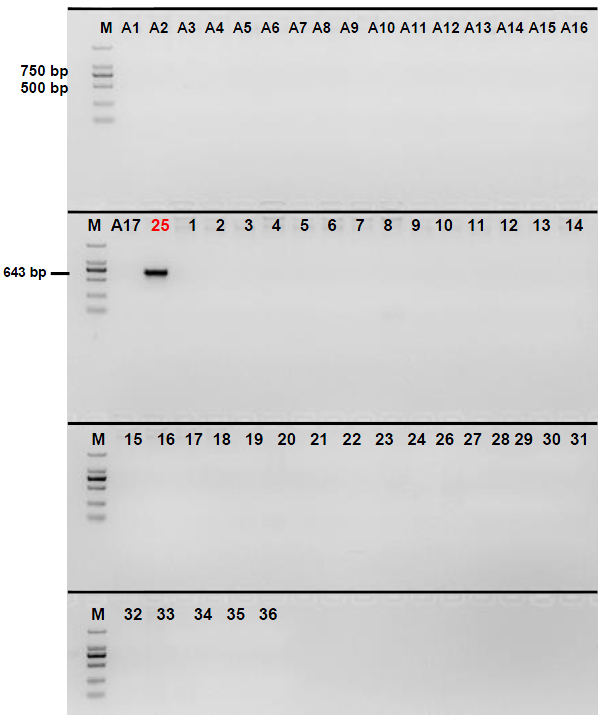
**Figure S6.** Validation of the 2.4 kb inversion in 54 animals. The founder animal #25 was marked with red color.

**Table S1** Detailed information of the three trios for whole-genome sequencing.

| Trio | Animal ID | Raw data (G) | Clean data (G) | Reads generated | Mapped sequences (%) | Sequencing  coverage |
| --- | --- | --- | --- | --- | --- | --- |
| *Trio_1* | ♂: #114989 | 54.77 | 53.94 | 539,385,340 | 536,057,692 (99.38%) | 26.98× |
|  | *♀*: #05537 | 57.48 | 56.67 | 566,692,058 | 564,529,062 (99.62%) | 28.87× |
|  | **Founder: #25** | 46.57 | 46.02 | 460,190,291 | 457,409,342 (99.40%) | 23.08× |
| *Trio_2* | ♂: #09331 | 57.48 | 49.12 | 491,233,819 | 487,998,654 (99.34%) | 24.57× |
|  | *♀*: #1202016 | 54.73 | 53.94 | 539,385,340 | 536,057,692 (99.38%) | 25.45× |
|  | **Founder: #28** | 54.37 | 53.75 | 537,473,735 | 534,835,112 (99.51%) | 26.88× |
| *Trio_3* | ♂: #11009 | 51.76 | 51.11 | 511,081,028 | 508,508,552 (99.50%) | 25.75× |
|  | *♀*: #1202016 | 54.73 | 53.94 | 539,385,340 | 536,057,692 (99.38%) | 25.45× |
|  | **Founder: #A9** | 51.18 | 50.43 | 504,326,144 | 502,107,171 (99.56%) | 25.74× |
